# Mitochondria, PPARs, and Cancer: Is Receptor-Independent Action of PPAR Agonists a Key?

**DOI:** 10.1155/2008/256251

**Published:** 2008-07-14

**Authors:** Roberto Scatena, Patrizia Bottoni, Bruno Giardina

**Affiliations:** Department of Laboratory Medicine, Catholic University, Largo A. Gemelli 8, 00168 Rome, Italy

## Abstract

Before the discovery of peroxisome proliferator activated receptors (PPARs), it was well known that certain drugs considered as classical PPAR-alpha agonists induced hepatocarcinoma or peroxisome proliferation in rodents. These drugs were derivatives of fibric acid, and they included clofibrate, bezafibrate, and fenofibrate. However, such toxicity has never been observed in human patients treated with these hypolipidemic drugs. Thiazolidinediones are a new class of PPAR activators showing greater specificity for the *γ* isoform of PPARs. These drugs are used as insulin sensitizers in the treatment of type II diabetes. In addition, they have been shown to induce cell differentiation or apoptosis in various experimental models of cancer. PPAR-*α* ligands have also been shown to induce cancer cell differentiation and, paradoxically, PPAR-*γ* drug activators have been reported to act as carcinogens. The confusing picture that emerges from these data is further complicated by the series of intriguing side effects observed following administration of pharmacological PPAR ligands (rhabdomyolysis, liver and heart toxicity, anemia, leucopenia). These side effects cannot be easily explained by simple interactions between the drug and nuclear receptors. Rather, these side effects seem to indicate that the ligands have biological activity independent of the nuclear receptors. Considering the emerging role of mitochondria in cancer and the potential metabolic connections between this organelle and PPAR physiology, characterization of the reciprocal influences is fundamental not only for a better understanding of cancer biology, but also for more defined pharmacotoxicological profiles of drugs that modulate PPARs.

## 1. INTRODUCTION

Since the discovery of the first PPAR by Issemann and Green in 1990 [1], the role of this fascinating class of nuclear receptors in normal physiology and pathophysiology has become progressively more important. The potential biological activities attributed to PPARs have been expanding ever since they were identified as potential mediators of the hypolipidemic effect of fibrates in humans and as participants in peroxisome proliferation and hepatocarcinogenicity in rodents. So far, PPARs have been implicated in diverse processes including lipid and carbohydrate metabolism, energy expenditure, immune and inflammatory processes, vascular homeostasis, tissue remodeling, and cell differentiation and proliferation in normal and neoplastic tissues [2–8].

It is evident, but too often ignored, that important interrelationships must exist among PPARs, mitochondria, and cancer. Regardless of the precise nature of these interconnections, PPARs undoubtedly have a significant energetic, plastic, and signaling role in the pathophysiology of cancer cells purely by virtue of their central role in lipid metabolism. This role necessarily involves interaction with mitochondria. Mitochondria are not only the main site of lipid oxidative metabolism, but they are also the cellular powerhouses that coordinate cellular metabolism and serve as the origin of important anabolic fluxes and signal transduction pathways [9, 10].

Thus, the role of mitochondria in cancer is under a critical re-evaluation, particularly in light of the so-called Warburg effect: most cancer cells exhibit increased aerobic glycolysis, and use this metabolic pathway for generation of ATP as a main source of their energy supply. Too often this does not justify the complex metabolic alterations present in different types of neoplasia [11–13].

Similarly, the interrelationships between PPARs and cancer are not entirely clear. Some studies show that PPARs have antineoplastic and/or cancer differentiating activities, while others show that they have important carcinogenic properties [6, 14–20].

These reports highlight the multifaceted role of PPARs in neoplastic cells. Hence, the roles of PPAR in normal physiology and pathophysiology should be clarified, since this may benefit our understanding of how cancer occurs and how it can be treated.

Discussion of the interrelationships among PPARs, mitochondria, and cancer should first involve careful evaluation of some misleading factors that have contributed to confusion about PPAR biology.

## 2. PPARs IN PATHOPHYSIOLOGY AND MISLEADING FACTORS

### 2.1. Synthetic ligands

Initially, the physiological ligands of PPARs were unknown and PPARs were classified as “orphan receptors.” Their function was studied using synthetic ligands of PPAR-*α*, the first PPAR discovered. These synthetic ligands were a heterogeneous class of molecules ranging from trichloroacetic acid to plasticizers such as di-2-ethylhexyl phthalate (DEHP) and mono-2-ethylhexyl phthalate (MEHP) [1]. The structural heterogeneity of the ligands seems to reflect the conformation of the PPAR ligand binding domain (LBD), which forms a large, Y-shaped hydrophobic pocket with relatively low ligand specificity [2, 7]. For PPAR-*α*, the ligands used most often in experiments were fibrates such as clofibric acid, bezafibrate, and gemfibrozil. Studies on fibrate binding to PPAR-*α* showed that these drugs caused a hypotriglyceridemic effect by inducing transcription of several genes related to oxidative metabolism of lipids, which occurs primarily in the mitochondria [2–4]. However, some discrepancies soon emerged. In fact, previous PPAR research failed to provide thorough explanations of the drugs' other important biological activities, such as peroxisome proliferation and hepatocarcinogenicity in rodents or, most importantly, their side effects. These side effects include angina crisis; elevation of serum aminotransferase, which indicates liver damage; increases in serum creatine phosphokinase concentrations, which can initiate myositis, myopathy and, in rare cases, rhabdomyolysis; increases in serum creatinine concentration; and acute renal failure in rare cases [21].

Some of these biological activities had already led investigators to propose that these ligands had effects independent of their binding to PPAR. If true, neglecting these “extrareceptor” functions may interfere with our understanding of PPAR pathophysiology. For example, studies in the 1980s established that fibrates (clofibric acid, bezafibrate, and gemfibrozil) can pass freely through red blood cell membranes and bind to human hemoglobin at the level of the hydrophobic pocket of the alpha chain interfaces. This binding lowers the oxygen affinity of hemoprotein more strongly than does the natural allosteric effector 2,3-biphosphoglycerate [22], which could, for example, lead to an angina crisis (by perturbation of microcirculation in ischemic areas caused by abrupt changes in the level of oxygen released from the blood) at the usual therapeutic plasmatic drug concentrations [23]. Around the same time, other researchers showed that the agents acting as peroxisome proliferators hampered mitochondrial respiration, with potentially significant clinical implications [24–26]. Indeed, treatment with fibrates was found to lead to some histological and biochemical features characteristic of hepatic, muscular, and renal toxicities. This result led to the hypothesis that disruption of the mitochondrial electron respiratory chain in conjunction with other genetic or acquired predisposing factors may contribute to these toxic effects independently of PPAR activation. Moreover, molecular analysis of the interactions between human hemoglobin and fibrates indicated that particular physicochemical aspects of the drug molecules, specifically their carboxylic group and their significant hydrophobicity, might be responsible for their biological activity. Interestingly, these physicochemical characteristics of fibrates fit well with the milieu of mitochondria in general, namely the difference in pH between the more alkaline matrix and the more acidic intermembrane space, as well as with structural features of complex I (NADH cytochrome c reductase) in the mitochondrial electron respiratory chain, which is a large, hydrophobic protein component in the mitochondria [10, 27].

**Figure 1:**
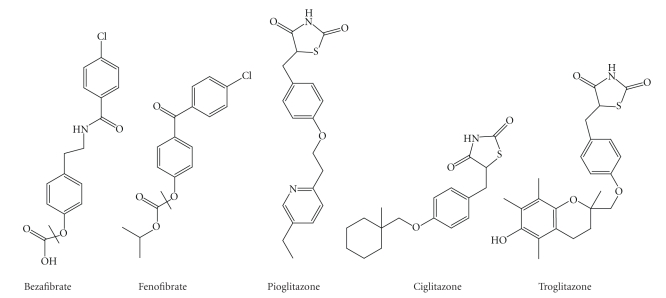
Structure of some drugs acting as PPAR ligands.

**Figure 2:**
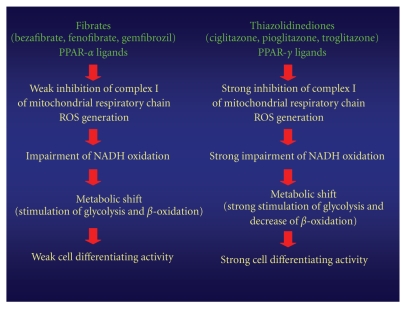
Flow chart of possible molecular mechanisms caused by a mitochondrial dysfunction induced by fibrates (PPAR-*α* ligands) and thiazolidinediones (PPAR-*γ* ligands).

All of these considerations led to studies that examined how mitochondria were affected by fibrate administration, and whether these effects might have clinical implications [18, 28]. The results clearly showed that fibrates could disrupt the mitochondrial electron respiratory chain at the level of NADH cytochrome c reductase [18]. This effect was even more pronounced for ciglitazone, which was one of the first thiazolidinediones to be synthesized. Thiazolidinediones are a class of molecules that are chemically related to fibrates (Figure 1). Drug-induced mitochondrial dysfunction causes a series of compensatory metabolic mechanisms, which, in addition to PPAR agonist activity, may be partially responsible for some of the pharmacological and toxicological properties of this class of molecules. In fact, the resulting shut-down of mitochondrial NADH oxidation drives cells to change their oxidative metabolism in a way that is strictly correlated to the degree of complex I inhibition. Specifically, upon treatment with fibrates, which are less potent inhibitors of complex I, cells tend to use those components of the electron respiratory chain that remain efficient (e.g., complex II). This leads the cell to use FADH_2_ oxidation to obtain energy. In other words, compensatory mechanisms come into play, which are probably sustained by glycerol catabolism viamitochondrial FAD-dependent glycerol-3-phosphate dehydrogenase or by fatty acid *β*-oxidation viaelectron-transferring flavoprotein (ETF). These changes have a significant hypotriglyceridemic effect and a slight hypoglycemic effect. In contrast, using more potent complex I inhibitors (i.e., thiazodilinediones) greatly reduces NADH dehydrogenase activity, thus reducing the use of *β*-oxidation and increasing reliance on glycolysis, resulting in a stronger hypoglycemic effect and a much weaker or null hypotriglyceridemic effect (Figure 2) [18].

Based on these findings, PPAR-*α* activation may be due, at least in part, to a shift in the metabolic state: preferential use of lipids through glycerol catabolism via mitochondrial FAD-dependent glycerol-3-phosphate dehydrogenase and fatty acid *β*-oxidation via ETF. In this way, as suggested by Kersten et al. [3], fatty acids would stimulate their own metabolism. Interestingly, this mechanism of switching to lipid metabolism in response to fibrate-induced complex I inhibition may explain the reported activation of genes in the cytochrome P450 IV family. This family of proteins is responsible for microsomal *ω*-oxidation of long chain and very long chain fatty acids [29]. This pathogenic mechanism may also provide a better explanation of the peroxisomal proliferation observed in rodents given synthetic PPAR-*α* ligands. In fact, the lipid component of these animals' diet contains a particularly high proportion of polyunsaturated fatty acids [30]. Hence peroxisomal *β*-oxidation, which is normally more active in rodents than in humans or other primates, may be further enhanced by inhibition of mitochondrial NADH oxidation.

Moreover, the increase in free radical oxygen species resulting from stimulated peroxisomal *β*-oxidation may further increase the oxidative stress that results from complex I inhibition [31, 32] and thereby contributes significantly to the observed carcinogenic properties of PPAR ligands in rodents, particularly in the liver.

From a clinical point of view, thiazolidinediones, which are stronger inhibitors of NADH cytochrome c reductase [18, 33–36] than fibrates, strongly disrupt NADH oxidation such that they may prevent the induction of *β*-oxidation, which could otherwise serve as a compensatory energy source. This renders metabolism almost exclusively dependent on glycolysis. Interestingly, damage to the pathways of energy production, particularly in organs that are rich in mitochondria (e.g., liver, muscle, heart, and kidney), may explain (i) the prevalent hypoglycemic activity of *γ*-ligands, despite minor or absent hypolipidemic effects, (ii) the weight gain due mainly to water retention typically observed in patients treated with PPAR-*γ* ligands, (iii) the differentiation of adipose cells, and (iv) the dramatic cardiac and hepatic toxicities often observed following administration of thiazolidinediones [37–40].

Interestingly, similar mitochondrial impairment by PPAR agonists was observed by Nadanaciva et al. [41], who used a phosphorescent oxygen-sensitive probe and an immunocapture technique to evaluate the mitochondrial respiration and the activity of individual oxidative phosphorylation complexes on isolated rat liver mitochondria. By this dual approach, the authors were also able to obtain a rank order of the mitochondrial toxicity of thiazolidinediones, fibrates, and statins. These results could be important as they suggest the possibility of a screening strategy to evaluate potential mitochondrial toxicity, reducing in such a way the incidence of clinical side effects.

Moreover, a novel mitochondrial target protein has recently been identified for the thiazolidinediones [42]. This protein, called mitoNEET, is an iron-containing outer mitochondrial membrane protein that seems to play a role in regulating mitochondrial oxidative processes. Recently, Wiley et al. [43, 44] showed that cardiac mitochondria isolated from mitoNEET-null mice demonstrated a reduced oxidative capacity, confirming that mitoNEET is a protein involved in the control of maximal mitochondrial respiratory rates.

These results underline the importance of carefully defining the direct interrelationships between pharmacological PPAR ligands and mitochondria, also to better clarify the physiology and pathophysiology of PPARs.

In conclusion, many PPAR ligands possess extrareceptor biological activities that can complicate interpretation of the results of experiments investigating the pathophysiology of PPARs. This caveat has serious consequences, not only for our ability to correctly understand the metabolic roles of PPARs, but also for our ability to determine their roles in the (de)differentiation of cancer cells.

### 2.2. Metabolic and genetic studies of PPARs in rodents

Most of the available data on the pathophysiology of PPARs has been obtained from metabolic studies in rodents. However, species-specific differences in metabolism and diet can be an obstacle when applying the results of animal studies to human patients [35, 45].

Another source of data that may be misleading is genetic studies on knock-out rodents for PPARs or their transcriptional coactivators (i.e., PGC-1*α*). Conclusions drawn from these studies about the metabolic roles of the different PPARs neglect the interaction between PPARs and their coactivators in mitochondrial biogenesis in general, and in mitochondrial lipid metabolism in particular [5, 46–49].

## 3. PPAR AND CANCER: WHAT IS THE ROLE FOR MITOCHONDRIA?

Various molecular links between PPARs and cancer have been considered in other articles. Here, we highlight some intriguing observations related to PPARs and cancer that seem to indicate a role for mitochondria in this disease of cell proliferation and differentiation. Since the aim of this review is to discuss the potential molecular link between PPARs and cancer from a mitochondrial point of view, we focus on the particular “extrareceptor” interrelationships between PPARs and fibrates or their thiazolidinedione derivatives.

### 3.1. PPARs, mitochondria, and carcinogenesis

PPARs and their pharmacological ligands were originally considered to be “oncopromoters.” Specifically, PPAR ligands were considered to be nongenotoxic carcinogens. It is well known that these molecules can induce hepatocarcinoma in rodents; however, their administration first provokes hepatomegalia and induces expression of a series of antioxidant enzymes, such as catalase, superoxide dismutase, and glutathione peroxidase. In this way, these molecules may create an imbalance in oxidative metabolism in general and oxidative stress in particular [50, 51]. The ability of PPAR ligands to induce oxidative stress has since been confirmed for thiazolidinediones as well [52–54]. Interestingly, reactive oxygen species (ROS) and cellular oxidative stress have long been implicated in carcinogenesis, despite the fact that the precise pathogenic molecular mechanisms are complex, debated, and at times paradoxical [55]. More specifically, the following hold.

- In normal cells, mutations in nuclear or mitochondrial genes encoding components of the mitochondrial electron transport chain (ETC) or xenobiotics capable of disrupting the mitochondrial electron flux can lead to an increase in the generation of ROS, particularly superoxide. This radical is rapidly dismuted by superoxide dismutase to yield hydrogen peroxide (H_2_O_2_), which can diffuse to the nucleus and attack DNA before cellular antioxidant defenses adjust to the new level of oxidative stress. This oxidative damage may contribute to genetic instability in congenital and/or acquired predisposed subjects [56, 57].
- Cancer cells generally generate more ROS than normal cells. This difference may relate to the greater number of metabolic and proliferative activities that often occur in a transformed cell, or to a qualitative or quantitative imbalance between cellular antioxidant defenses and the oxidative environment [55, 58–60].
- In cancer cells, the levels of expression of some components of the antioxidant system are amplified independently by drug treatment (e.g., thioredoxin, DJ-1 protein, peptidyl-prolyl cis-trans isomerase A, cyclophilin A, protein disulfide isomerase A3, ERP 60/GRP58) [55, 61]. Interestingly, the increase in thioredoxin activity in cells with elevated oxidative stress may relate to its essential role in facilitating transcription in an environment where increased oxidative stress signaling in the cytosol is required for stimulating cell proliferation. Furthermore, drug-induced cancer cell differentiation typically reduces the expression of these antioxidant proteins [62].
- Numerous studies implicate increased oxidative stress in the cell death induced by diverse chemotherapeutic agents. Anthracycline derivatives, newer redox cycling agents, and, more recently, histone deacetylase inhibitors and proteasome inhibitors all appear to increase oxidative stress in cells. Although the mechanism responsible for the increase has not been established, mitochondria are fundamental in ROS generation and seem to be involved, either directly or indirectly [55, 63–65].

This puzzling picture suggests that PPAR-related rodent hepatocarcinogenesis depends on strong stimulation of ROS generation, mainly in mitochondria dysregulated by the PPAR ligand in question. Moreover, this oxidative stress may be reinforced by the specific membrane composition and the abundant H_2_O_2_ production from peroxisomal lipid metabolism in rodents, as already discussed.

Could a similar pathogenic mechanism have a role in human carcinogenesis?

A direct role for PPARs in carcinogenesis is hardly credible, considering their fundamental physiological role in cell metabolism. The altered expression of different PPAR isoforms observed in some neoplasias may be the result of secondary metabolic changes in transformed cells relative to normal cells [6, 66].

On the other hand, the question of whether the synthetic PPAR ligands play a role in human carcinogenesis is still open and intriguing. In fact, one of the first large clinical studies on gemfibrozil, a classic PPAR-*α* ligand, [67] showed a small but significant increase (*P* = .032 by the Fisher exact test) in the incidence of basal cell carcinoma in patients taking gemfibrozil; this finding, unfortunately has been largely ignored by investigators. Moreover, in an intermediate follow-up study [68], cancer occurred at equal rates in both the untreated group and the group treated with gemfibrozil, but the cancer in the latter led more often to mortality, primarily during the last 1.5 years of follow-up. To be sure, results recently obtained from an 18-year mortality follow-up of this study do not seem to confirm this increase in cancer mortality, but the follow-up design failed to address certain possible interpretations and also in the Autors opinion of this cited study some of the follow-up data can be misleading [69].

Nevertheless, the data obtained in the original study are intriguing considering the peculiar molecular epidemiology and pathogenesis of basal cell carcinoma and the relatively short (5-year) period of drug exposure used in the study. These findings, together with the demonstrated ability of fibrates and thiazolidinediones to alter mitochondrial oxidative metabolism and induce ROS generation, indicate that care should be taken when this class of drugs is used in the treatment of nutrition-sensitive tumors [70, 71].

### 3.2. PPARs, mitochondria, and inhibition of tumor growth

It is well established that activation of PPARs (*α*, *β*/*δ*, and *γ*) by natural or synthetic agonists can inhibit growth and induce differentiation or death of tumor cells. The original observation was of PPAR-*γ* ligands and liposarcoma, consistent with the physiological function of PPAR-*γ* [3, 72, 73]. Subsequently, PPAR-*γ* and PPAR-*α* ligands were shown to promote the differentiation of various tumor cell lines, including breast, lung, prostate, leukemia, colon, melanoma, and liver. This differentiation was often independent of the relative expression levels of the different PPAR isoforms [6, 15, 16, 66, 70, 72]. These studies also suggested extrareceptor activities of fibrates and thiazolidinediones as the basis of their ability to induce cancer cell differentiation [15–18, 72]. Moreover, a recent study by Panigrahy et al. [74, 75] on endothelial and mesenchymal tumor cells and mice showed that PPAR-*α* ligands such as fenofibrate directly suppress tumor growth through receptor-dependent and -independent pathways, and that they indirectly suppress tumor growth by inhibiting angiogenesis and the inflammatory response in the microenvironment of the tumor. Therefore, the noncancerous host tissue could be an important target for cancer treatment with pharmacological PPAR ligands.

These data illustrate the extreme complexity of the interrelationships among PPARs, mitochondria, and cancer. Nevertheless, the most important aspects of these interrelationships are the activities of the synthetic PPAR ligands, particularly their extrareceptor activities. Mitochondria are becoming increasingly important as targets for these drug-induced extrareceptor activities, as discussed in recent reviews [35, 76, 77]. To better understand the interactions among PPAR ligands, mitochondria, and cancer, it may be useful to describe our work, which parallels that of other groups. Curiously, the differentiating activity of fibrates was originally hypothesized in binding studies of fibrates and hemoglobin. The physicochemical properties of fibrates and their toxicological profile allow them to interact with some hydrophobic components of the mitochondrial electron respiratory chain. The resulting oxidative metabolic stress may induce differentiation of cancer cells, similar to the effects of heat shock [15]. Importantly, this effect does not depend on PPAR agonism, but it is related to the physicochemical properties (pKa, log P, log D, water solubility, and pH profile) of the molecules. These properties should favor permeation, accumulation, and interaction with components of the internal mitochondrial membrane [10].

For example, therapeutic doses of bezafibrate inhibited proliferation of human leukemia cell lines HL-60, U-937, and K-562 in a dose-dependent manner. In HL-60 cells, growth inhibition was associated with an increased number of cells in the G0/G1 phase and a significant decrease in the number of cells in the G2/M phase. Analysis of cell differentiation markers (CD) showed a dose-dependent increase in expression of CD11b and CD14 in HL-60 cells and of CD14 in U-937 cells. Functional assays confirmed that the phenotypes of these cells were more mature. Both HL-60 and U-937 cells showed a dose-dependent restoration of the respiratory burst stimulated by PMA and zymosan. K-562 erythroleukemia cells showed a dose-dependent increase in hemoglobin synthesis. Similar cellular differentiation was observed following treatment with two other fibrate derivatives, clofibric acid and gemfibrozil. Interestingly, fibrate-induced differentiation was partially inhibited by antioxidants including acetylcysteine (NAC), and electron microscopy revealed that fibrate-treated cells had mitochondrial damage [15]. Functional evaluation of this drug-induced mitochondriopathy showed that fibrates and ciglitazone specifically inhibited NADH cytochrome c reductase activity in a dose-dependent manner in HL-60, TE-671 human rabdomyosarcoma, and Hep-G2 human hepatocarcinoma cell lines, whereas the activity of other mitochondrial respiratory chain enzymes remained unchanged [18, 33, 36]. The impairment of NADH oxidation induced a cellular metabolic shift towards anaerobic glycolysis and/or *β*-oxidation, as shown by the dose-dependent increases of certain metabolites (lactate, alanine, glycolytic, and nonglycolytic derived acetate) [18].

A fundamental observation from this research was the correlation of mitochondrial dysfunction, metabolic shift, and differentiation activity in tumor cells treated with increasing concentrations of PPAR ligands. Furthermore, quantitative comparison on a molar ratio basis between these PPAR ligands (bezafibrate, clofibric acid, gemfibrozil, and ciglitazone) for inhibition of NADH cytochrome c reductase activity, metabolic adaptations, differentiation potency, and antiproliferative index confirmed a strict correlation between these parameters [18]. These results suggested that inhibition of mitochondrial NADH dehydrogenase could contribute to both the pharmacological and toxicological profiles of fibrate derivatives (strong hypolipidemic/weak hypoglycemic effect, liver and muscle toxicity) and thiazolidinediones (hypoglycemic/insulin sensitizer effect, liver and heart toxicity) [17, 18, 25, 26, 33–40].

In terms of mitochondrial oncology, these data suggest a possible molecular mechanism for the peroxisome proliferator activity and carcinogenicity of fibrates typically observed in rodents. These data also indicate a strict correlation among fibrate- and thiazolidinedione-induced cellular respiration dysfunction, stimulation of glycolysis, and cancer cell differentiation that strongly implicates mitochondria and oxidative metabolism in the pathophysiology of cancer.

Importantly, these results confirm and extend the results of other studies focusing on nongenomic activities of fibrates and thiazolidinediones [16, 17, 78–81]. Furthermore, these observations explain some contradictory data related to the role of PPARs in cancer cell differentiation [3, 6, 72, 77]. Above all, the intriguing data concerning the induction of differentiation associated with a shift towards aerobic glycolysis (a paradoxical Warburg effect) confirms the need to reconsider cancer cell metabolism in general and the Warburg effect in particular [11, 13, 82]. To that end, our understanding of the role of PPARs in cancer should assume a new level of complexity that takes into account their fundamental functions in lipid metabolism, in inflammation and, directly or indirectly, in angiogenesis [74, 75].

The molecular link among the synthetic PPAR ligands, mitochondria, and cancer indicates the need for a careful evaluation of some aspects of cancer cell pathophysiology, such as the following.

- The possible existence of a transduction pathway master signal as the basis of the complex cellular differentiation program related to PPAR. ROS, nitric oxide (NO), and reactive nitric oxide species (RNS) should form an important branch of this program. In addition, there should be a role for the NADH/NAD+ ratio.
- The role of some oncogenes/oncosuppressors in cancer pathogenesis, given that mitochondrial respiratory chain dysfunction can induce a more differentiated phenotype in tumor cells and thereby influence their activity [15–18].
- The significance of the modulation of the expression of proteins with oncogenic and antioxidant functions (stathmin 1, DJ-1 protein, peroxiredoxin 2, nucleoside diphosphate kinase A, etc.) in PPAR-related cancer cell differentiation. This is an important topic given the potential pathophysiological role of PPAR in cancer [62, 83].

At last, an understanding of the molecular mechanisms involved in the interrelationships between mitochondrial respiration and PPAR-related cancer regression may have important clinical implications for cancer diagnosis, prognosis, and therapy.

## 4. CONCLUSION

Many molecular mechanisms have been proposed to explain how PPARs, directly and/or indirectly, may induce cancer cell cycle arrest and induction or cancer cell differentiation or dedifferentiation. In spite of this, the molecular interrelationships between the mechanisms of functional modulation of PPAR and these important cellular phenotypic changes are still debated. It is clear that the various molecular modifications observed in different studies (decrease in cyclin D1, inhibition of IkB, induction of TSC22, NF-kB, GADD153, PTEN, etc.) may depend on the particular cell and cell functional status and that a potential master signal should be investigated.

Here, we have briefly described the molecular link between PPARs and cancer from a mitochondrial point of view. In our opinion, the most important factor linking cancer to PPARs is represented by their “synthetic ligands,” which are characterized by other important and debated extrareceptor activities. Specifically, these agents can induce oxidative stress, which has an ambiguous role in cancer, leading it to act as a double-edged sword.

In this sense, mitochondria plays a critical role as one of the most important organelles for generating reactive species. The metabolic stress and energetic failure that result from fibrate- and thiazolidinedione-induced mitochondrial impairment may also play an important part in cancer regression, especially in cells that require active and complete anabolic pathways to sustain cancer growth, an aspect that has not always been completely or correctly evaluated.

Considering their physicochemical properties discussed above, it is worthy of note that tumor regression induced by PPAR ligands may be a useful approach for the treatment of neoplasias of the central nervous system, which are classically difficult to treat with conventional chemotherapy. Specifically, interesting results have already been obtained in terms of decrease of cell proliferation, apoptosis induction, and expression of markers typical of a more differentiated phenotype in glioblastoma and astrocytoma cell lines [84–90], in primary cultures of human glioblastoma cells derived from surgical specimens [91], and above all, in patients with high-grade gliomas (glioblastoma or anaplastic glioma) [92].

Moreover, given that pharmacological modulation of PPAR in cancer cells typically arrests the cell cycle in the G0/G1 phase, combination therapy with a PPAR agonist and an antimitotic antitumor agent deserves careful consideration.

Furthermore, it may be useful to distinguish between real differentiating agents, which show low cytotoxicity indices relative to their differentiation activity (thiazolidinediones, fibrates, retinoids) and spurious differentiating agents, which show low differentiating activity and high cytotoxicity indices (old and new HDAC inhibitors) [93, 94].

A mitochondrial approach to analysis of the molecular link between PPARs and cancer certainly adds new levels of complexity to the already complicated picture. However, an optimal definition of all molecular mechanisms relating PPARs, mitochondria, and cancer may be fundamental to our understanding of the real therapeutic index of pharmacological modulation of these nuclear receptors. This is important not only in cancer, but also in the other diseases in which PPARs play a significant role, including atherosclerosis, hyperlipoproteinemias, metabolic syndrome, diabetes mellitus, and obesity. Moreover, a complete understanding of the pharmacotoxicological profile of these agents may reduce the incidence of dangerous side effects that have already dramatically afflicted patients treated with PPAR ligands [37–40].
